# Predictability in Cellular Automata

**DOI:** 10.1371/journal.pone.0108177

**Published:** 2014-10-01

**Authors:** Alexandru Agapie, Anca Andreica, Camelia Chira, Marius Giuclea

**Affiliations:** 1 Bucharest University of Economic Studies, Department of Applied Mathematics, Bucharest, Romania, and Institute for Mathematical Statistics and Applied Mathematics, Bucharest, Romania; 2 Babes-Bolyai University, Faculty of Mathematics & Informatics, Cluj-Napoca, Romania; 3 Bucharest University of Economic Studies, Department of Applied Mathematics, Bucharest, Romania; Universidad de Zarazoga, Spain

## Abstract

Modelled as finite homogeneous Markov chains, probabilistic cellular automata with local transition probabilities in (0, 1) always posses a stationary distribution. This result alone is not very helpful when it comes to predicting the final configuration; one needs also a formula connecting the probabilities in the stationary distribution to some intrinsic feature of the lattice configuration. Previous results on the asynchronous cellular automata have showed that such feature really exists. It is the number of zero-one borders within the automaton's binary configuration. An exponential formula in the number of zero-one borders has been proved for the 1-D, 2-D and 3-D *asynchronous* automata with neighborhood three, five and seven, respectively. We perform computer experiments on a *synchronous* cellular automaton to check whether the empirical distribution obeys also that theoretical formula. The numerical results indicate a perfect fit for neighbourhood three and five, which opens the way for a rigorous proof of the formula in this new, synchronous case.

## Introduction

From a mathematical point of view, *cellular automata* (CA) are binary lattices that are updated iteratively. In the automata discussed in this paper, the value of a cell is flipped based only on the number of ones in the neighborhood of the cell to be updated. We call an automaton *synchronous* if all cells are updated simultaneously, respectively *asynchronous* if the updating affects only one cell at a time.

We further call an automaton *deterministic* [5], [6], [17] if the update follows deterministic rules, respectively *probabilistic* [1]-[4], [7], [8], [11], [12] if at least one of the following holds:

• the updated cell is picked at random

• the local transition rule is probabilistic - e.g., a cell may flip from zero to one with some probability 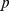, and the same cell may stay in zero with probability 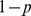.

Probabilistic automata are suitable for Markov chain modelling, since the future configuration of the automaton depends only on its present state.

A finite homogeneous Markov chain is a stochastic process that moves according to some probabilities within a finite set of states, say 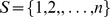, with transition probability from state 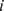 to state 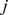 (denoted 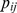) depending only on states 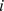 and 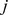. The square, non-negative *transition matrix* 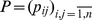 gathers all the above transition probabilities. Transition matrix of a Markov chain is always *stochastic* - that is, the sum of probabilities in each row is one, and since in our case the matrix does not change from an iteration to another, it is called *homogeneous*.

A brief introduction to homogeneous Markov chains is given in the following. For more detail, reader is referred to monographs [10], [13], [14].

**Definition 0.1.** • *A state* 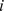 *is* absorbing *if* 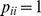. *An absorbing state is never left, once it is entered.*

• *A stochastic matrix* 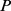 *is* primitive *if there is a positive integer* 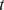 *such that* 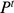 *is (strictly) positive.*

*• A stochastic matrix is called stable if all its rows are identical.*

• *Let* 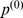 *be a probability vector. If* 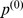 *is the initial distribution of the Markov chain with transition matrix* 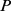, *then the distribution after* 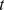 *steps is* 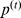, *with* 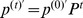, *for all* 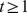. *If* 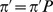, *then* 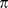 *is a* stationary distribution.

**Theorem 0.2.** *Let* 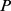 *be a* primitive *transition matrix. Then* 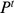 *converges as* 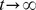 *to a positive stable stochastic matrix* 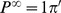, *and the rate of approach to the limit is geometric. Moreover, the limit distribution* 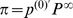 *has the following properties:*

• *is unique regardless of the initial distribution* 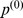;

• *has positive entries on all components;*

• *is also the unique stationary distribution of the associated Markov chain.*

There are many problems of interest in Markov chain theory [13]. The *short-term behavior* implies the correct definition of the transition matrix to be associated to some process. The *long term-behavior* is even more important, opening the way for prediction; that is strictly connected to the stationary distribution, and to finding necessary and sufficient conditions that guarantee its existence. Providing the stationary distribution in elegant, analytical form would be a bonus - fortunately, this is the case in our study. Finally, estimating the time the chain takes until convergence is also of interest - this topic is usually referred to in literature as *absorption time*.

When it comes to CA, literature has focused so far only on the first two topics. The computation of absorption time is also of certain interest, at least for the class of deterministic automata with two attractors, *all zeros* and *all ones*.

## Deterministic Cellular Automata

The monograph of Wolfram [17] and the papers of Chua and co-workers [5], [6] are referential works in deterministic CA literature. While Wolfram's pursuit of explaining complexity uses the empirical analysis of automata as a vehicle, Chua and co-workers put on mathematically sound clothes to Wolfram's original approach, in form of nonlinear differential equations [15], [16]. To make things clear, let us see first a particular CA at work.

Consider a two-state CA (the values of the states are set to 0 and 1 within this paper) with 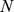 cells 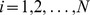 and *circular connection* - the left-hand neighbor of cell 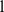 is cell 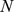. Cell 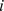 is influenced only by itself and its nearest neighbors 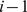 and 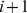. The values of cells 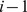, 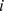 and 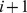 are the input of the process that is going to change cell 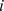. Such system is called 1-D three-neighborhood CA.

In the deterministic case, each of the eight possible input configurations 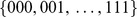 yields a certain output for the central cell 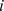. There are 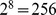 different functions 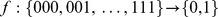 and each of these functions will be assimilated to a *local rule*. If we denote 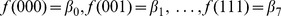, we have a one to one mapping between the 256 functions and the set of Boolean vectors 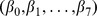. It is thus natural to identify each of the 256 functions by its associated decimal representation [5]

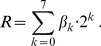
(1)


For example, the famous rule **110** - proved to be a *universal Touring machine* [17] - is defined synthetically by the Boolean vector 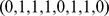, and explicitly by table 1. Notice that input consists of the whole three-neighborhood, while output is the new value of the central cell.

**Table 1:** Transition table for CA local rule 110.

Input	0**0**0	0**0**1	0**1**0	0**1**1	1**0**0	1**0**1	1**1**0	1**1**1
Output	**0**	**1**	**1**	**1**	**0**	**1**	**1**	**0**

It is hard to find an intuitive interpretation of rule **110**. Indeed, neither majority, nor minority governs the CA in table 1: 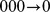 would indicate a majority rule, while 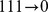 points otherwise.

That is not the case with rule **232**, which clearly defines a majority decision model, table 2.

**Table 2:** Transition table for CA local rule 232.

Input	0**0**0	0**0**1	0**1**0	0**1**1	1**0**0	1**0**1	1**1**0	1**1**1
Output	**0**	**0**	**0**	**1**	**0**	**1**	**1**	**1**

While Wolfram studied the 256 rules empirically, by running extensive computer experiments [17], Chua and co-workers proved rigorously that more insight into CA dynamic behavior can be gained by associating local rules like the one above to the attractors of so-called *cellular neural networks* (CNN).

As introduced in [5], CNN is a finite string 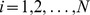 with circular connection, and a nonlinear dynamical system acting on each cell 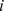, defined by a *state equation*

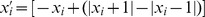


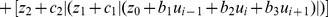
(2)

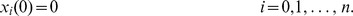
and an *output equation*

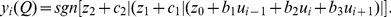
(3)


Equation (3) provides the steady state output Q of cell 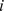 for each neighborhood input of type 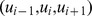, yet with symbol ‘0’ replaced by ‘-1’, e.g. 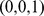 becomes 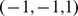. For each deterministic local rule, one can set the parameters 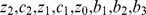 in the above equations such that the trajectory converges to an attractor Q with output 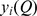 generating the -1/1 correspondent of the Boolean vector 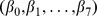 associated to the rule itself. For example, the state and output equations in case of rule **110** read
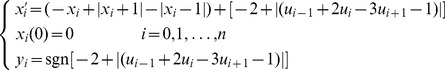
(4)


Equations (2)-(3) define only one CA iteration. According to [5], one can use a CNN chip to simulate ‘physically’ a local rule on all cells simultaneously. Therefore, one can describe each local rule as a nonlinear *difference equation*

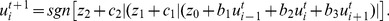
(5)


In case of majority rule **232** the difference equation simplifies to
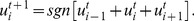
(6)


In [17], Wolfram starts from a fixed initial initial configuration 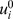, runs the 61-cell deterministic CA for each of the 256 local rules, independently, stores the produced configurations in large 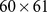 bi-colour arrays, then looks for similar patterns among arrays corresponding to different rules. He proves that rule **110** is universal Touring machine and, based on the geometrical similarity, conjectures that three other rules, namely **124**, **137** and **193** are also universal Touring machines.

Using Felix Klein's *Vierergruppe V*, Chua and co-workers obtain a classification of the 256 rules into 89 global equivalence classes [5]. Rules **110**, **124**, **137** and **193** fall into the same class, which gives a rigorous proof to Wolfram's conjecture. From a nonlinear dynamics point of view, these four rules are identical. As for the majority rule **232**, it forms a class by its own, there are no other rules equivalent to it. Another interesting application of this analysis is to the problem of *density classification*, see e.g. [9].

## Probabilistic Cellular Automata

In order to describe a probabilistic CA consider the 1-D three neighborhood automaton from the previous section, but with some randomness added to the local transition rule.

Consider first the model of an *asynchronous* CA - only one cell is flipped (at most) per iteration. We pick the cell for the flip uniformly - each cell with equal probability 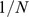. Once selected, the value of cell 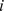 changes according to some local probabilities, depending on the number of ones within the significant neighborhood 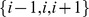, see table 3, where 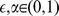 are the two parameters of the model.

**Table 3:** Local transition probabilities, 1-D three-neighborhood CA.

No. of ones	Probability 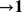	Probability 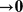
0	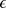	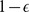
1	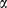	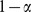
2	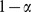	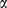
3	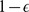	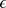

Table 3 considers all possible transitions, even the virtual ones. For instance, if value of cell 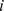 is 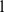 and there are two ones in the current neighborhood, cell 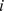 will ‘transit’ to 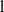 with probability 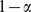. In other words, transition 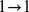 is still considered a flip. Compared to Wolfram's model of deterministic CA, the probabilistic model of table 3 allows for a unitary interpretation of local rules. Indeed, it makes no difference between local configurations 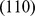 and 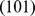 as they both have two ones, yet that does not mean that the middle cell will transit to the same value - it still depends on randomness.

The Markov model of the asynchronous three-neighborhood automaton has been introduced in [4]. There are 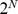 states in the Markov chain, consisting of all binary configurations of length 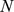. For an arbitrary state 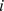 - here 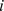 denotes a CA configuration, not a single cell - there are precisely 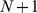 positive entries in row 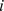 of the global transition matrix, namely the (global) transitions to states that differ from 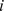 on a single cell, plus the element on the main diagonal.

The off-diagonal probabilities 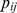 take values from the set 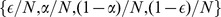 depending on the 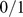 distribution of cells in the significant neighborhood of the cell in 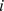 that should undergo a flip to get 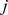. The diagonal probability 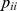 is equal to one minus all off-diagonal probabilities in row 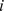. Since 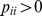 for 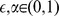, transition matrix is primitive. Then theorem 0.2 guarantees the existence of the *limit distribution*, also the (unique) *stationary distribution* of the Markov chain.

We found formulas for the stationary distribution of various asynchronous cellular automata [1], [3], [4], and we connected our findings to existent results from Ising and exponential voter model [2]. The most important results are presented in the following.

**Definition 0.3.** *A border occurs in a CA configuration between two different successive cells, like in 01 or 10. The total number of borders within configuration* 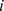 *is denoted* 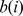.

Next theorem induces a class property on the set of configurations, revealing the stationary distribution as function of the number of borders.

**Theorem 0.4.** *The stationary distribution of transition matrix* 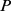 *of asynchronous three-neighborhood CA is* 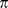, *whith*

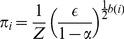
(7)
*and* 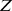 *a normalization factor.*

The computation of 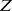 is solved by the following.

**Lemma 0.5.** *The number of configurations with* 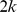 *borders is* 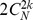, *for all* 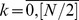.

Before moving further let us explain the practical meaning of the above results. The stationary distribution of the automaton is a probability vector with strictly positive components. That means the CA will not converge to a single state, but it will journey through all states, the sojourn time of each state being proportional to the corresponding probability in the stationary distribution. The succession of states in the journey remains unpredictable. What we can predict is that some configurations will have larger sojourn times than others, and formula (7) maps the sojourn time of a configuration to an exponential function of the number of borders within that configuration. Lemma 0.5 shows how many configurations fall in each class. Within a particular class, all configurations have exactly the same probability in the stationary distribution, thus their sojourn times will be the same.

It is also worth mentioning that the initial CA configuration does not influence the long term behavior of the probabilistic automaton, since the stationary distribution is independent of the Markov chain's starting point.

The *majority* model fulfils 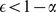, so the basis of the exponential function (7) is sub-unitary, and the larger the number of borders within a configuration, the smaller the time spent by the automaton in that configuration. Consequently, configurations *all zeros* and *all ones*, which both belong to class 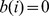, have the largest sojourn times, while configurations 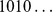 and 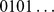 (with maximal number of borders) have the smallest sojourn times. Needless to say, situation reverses completely if 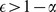.

In case of the five-neighborhood CA, there is one more parameter involved, call it 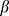, table 4, and the generalization of theorem (0.4) requires a supplementary condition on 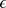, 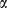 and 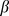, which ensures the so-called *detailed balance equation*.

**Table 4:** Local transition probabilities, 1-D five-neighborhood CA.

No. of ones	Probability 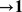	Probability 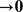
0	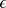	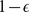
1	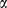	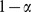
2	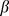	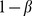
3	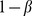	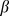
4	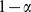	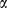
5	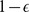	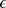

A refinement of the border definition is first needed.

**Definition 0.6.** *A* k-border *occurs between two different cells situated at distance* 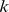 *from each other. E.g., in* 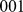 *we have a* 2-border *between first and third cell, and a* 1-border *between second and third cell.*

**Theorem 0.7.** *If the following holds*

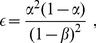
(8)
*then the stationary distribution of transition matrix* 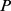 *of asynchronous five-neighborhood CA is* 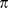, *with*

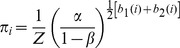
(9)and 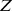 a normalization factor.

An analogous of lemma 0.5 also holds.

**Lemma 0.8** *The number of configurations with* 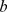 *order-1 borders and* 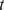 *order-2 borders is*

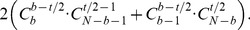
(10)


## Numerical Simulation

Using computer experiments we test the generality of the stationary distributions from previous section. Our assumption is that *synchronous* automata obey the same probability laws as their *asynchronous* counterparts. In order to build a synchronous CA, we drop the ‘only one cell per iteration undergoes a flip’ condition, and update all the cells in the same iteration, one by one from left to right.

For each numerical simulation we run 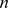 CA iterations starting from an arbitrary configuration and store the next 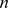 iterations in order to build an *empirical stationary distribution* - 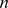 ranges between 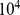 and 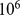.

Consider the three-neighborhood automaton. Formula (7) stands for the theoretical distribution, with constant 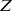 provided by lemma 0.5. We set local probabilities to 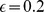 and 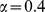, and the length of CA to 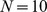; that yields the following partition of the configuration space w.r.t. the number of borders: 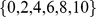.

**Figure 1:**
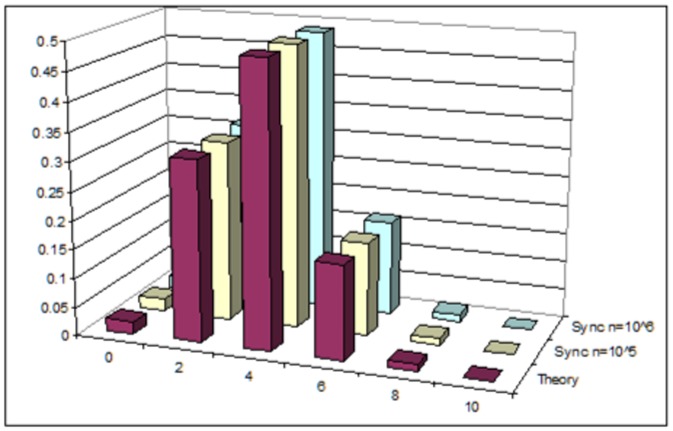
Three-neighborhood stationary distribution: Theoretical vs. empirical.

Figure 1 shows a perfect match between the theoretical distribution and the empirical distribution of asynchronous CA, with 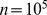 and 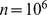, respectively. The value of 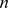 has no influence on the numerical results.

We consider next the five-neighborhood CA. The following lemma explains the partition of the configuration space in this case.

**Lemma 0.9.** *The partition induced by formula (10) on the five-neighborhood CA with length* 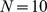 *is given in* *table 5* *.*

**Table 5:** State partition for five-neighborhood synchronous CA, 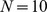.

Order-1 plus order-2 borders	0	4	6	8	10	12	14
No. of configurations	2	20	90	170	372	320	50

*Proof.* We need to consider all possible cases w.r.t. the sum of order-1 and order-2 borders, and for each case we should count the configurations with formula (10). Notice that the number of borders is always even, regardless of the order.

*Class 0*

There are only two configurations in this class, namely *all zeros* and *all ones*.

*Class 2*

One can easily check that this class is empty: there is no configuration with 2 order-1 and 0 order-2 borders, nor vice versa.

*Class 4*

The only non-void combination of borders in this class is 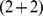, that is, 2 order-1 and 2 order-2 borders. Formula (10) provides in this case
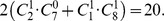



*Class 6*

The only cases in this class are 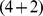 and 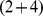, for which we compute
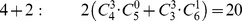


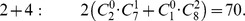



*Class 8*

The only cases in this class are 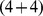 and 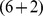, for which we compute
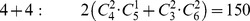


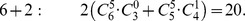



*Class 10*

The only cases in this class are 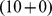 - for which there are only two configurations, namely *0101010101* and *1010101010*, respectively 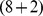, 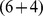 and 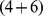, for which we compute
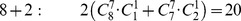


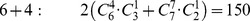


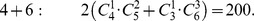



*Class 12*

The only cases in this class are 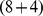, 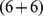 and 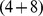, for which we compute
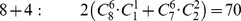


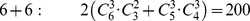


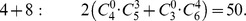



*Class 14*

The only case here is 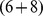, for which we compute
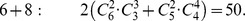



Summing up the number of configurations in each class completes the proof.

**Figure 2:**
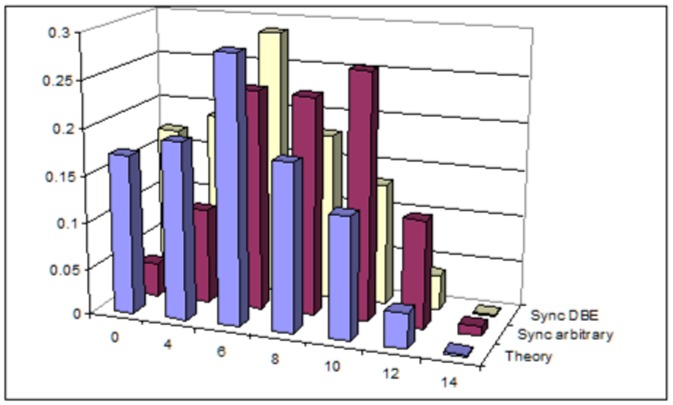
Five-neighborhood stationary distribution: Theoretical vs. empirical with arbitrary parameters, res. parameters from equation (DBE).

Basically, we performed the same tests as for the three-neighborhood automaton, with 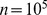. There is a difference, though. Theorem 0.7 provides the exponential form of the stationary distribution, but only under condition (8), which ensures detailed balance equation for the associated Markov chain. So it makes sense to test numerically whether this condition is really necessary. We present below results for the five-neighborhood synchronous CA, under two different settings of local transition probabilities: one *arbitrary*, 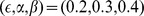, not fulfilling (8), and the other, 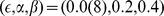, in perfect agreement with (8) and denoted *DBE* in figure 2. For *Theory* we used the theoretical stationary distribution (9) of the asynchronous case. The fact that the empirical distribution of the automaton with arbitrary local probabilities is far from the theoretical formula proves that condition (8) can not be removed.

## Discussion

Classification and prediction are the key issues in cellular automata. In the deterministic case, Wolfram relied on the inspiration of a computer analyst to derive patterns from the experimental simulation of different local rules. Chua and co-workers took the analysis a step further by demonstrating rigorously that every local rule can be mapped to a nonlinear dynamical system whose attractors encode accurately that very rule.

The situation is different with Markov chains. Here, predictability takes the form of the stationary distribution, which gathers the long-term sojourn times of each and every state of the system under consideration. For large systems like cellular automata, the existence of the stationary distribution alone is not very helpful, unless we have also an analytic formula able to connect the probabilities in the stationary distribution to some intrinsic features of the automaton configurations. Such fortunate situation is demonstrated in the paper, with an exponential stationary distribution, function of the number of borders within the configuration. The formulas, rigorously proved in previous papers for the asynchronous case, have been successfully tested via computer simulation on synchronous automata.

So far, the validation is only numerical, but the very good agreement between the (theoretical) formula and the (empirical) stationary distribution of synchronous automaton is a clear indication of the generality of the formula. As usually the case in theoretical computer science, the experimental results open the way for rigorous mathematical proofs, as well as for enlarging the test-bed by considering different variants of cellular automata. Another direction for future research is the stochastic analysis of absorption time, in case of the automata converging to the extreme configurations *all zeros* and *all ones*.

## Supporting Information

File S1 **Numerical results for different automata.**
(XLS)
